# Photo-Response of Functionalized Self-Assembled Graphene Oxide on Zinc Oxide Heterostructure to UV Illumination

**DOI:** 10.1186/s11671-015-1221-8

**Published:** 2016-01-12

**Authors:** A. N. Fouda, A. B. El Basaty, E. A. Eid

**Affiliations:** Physics Department, Faculty of Science, Suez Canal University, Ismailia, 41522 Egypt; Recruitment Department, University of Hail, Hail, 2440 Kingdom of Saudi Arabia; Basic Science Department, Faculty of Industrial Education, Helwan University, Cairo, Egypt; Department of Basic Science, Higher Technological Institute, 10th of Ramadan City, Egypt

**Keywords:** Structure modeling, Graphene oxide on ZnO, Self-assembly, Raman analysis

## Abstract

Convective assembly technique which is a simple and scalable method was used for coating uniform graphene oxide (GO) nanosheets on zinc oxide (ZnO) thin films. Upon UV irradiation, an enhancement in the on-off ratio was observed after functionalizing ZnO films by GO nanosheets. The calculations of on-off ratio, the device responsivity, and the external quantum efficiency were investigated and implied that the GO layer provides a stable pathway for electron transport. Structural investigations of the assembled GO and the heterostructure of GO on ZnO were performed using scanning electron microscopy (SEM), transmission electron microscopy (TEM), X-ray diffraction (XRD), and Fourier transform infrared spectroscopy (FTIR). The covered GO layer has a wide continuous area, with wrinkles and folds at the edges. In addition, the phonon lattice vibrations were investigated by Raman analysis. For GO and the heterostructure, a little change in the ratio between the D-band and G-band was found which means that no additional defects were formed within the heterostructure.

## Background

Much attention has been attracted to the coupling of graphene oxide (GO) and graphene (GR) with some semiconductors, which makes a proper enhancement in the charge transport, photocatalytic activity, and thermal conductivity [1–4]. In particular, GO/ZnO heterostructure is desirable for the inverted structure of hybrid solar cells [5], transparent electrode in optoelectronic devices [6], photocatalytic active devices [7], and sensors [8]. Zinc oxide (ZnO) has a large exciton binding energy of 59 meV, wide band gap of 3.37 eV at room temperature, piezoelectricity, catalytic activity, low cost in production, and bio-compatibility and is non-toxic (environmental friendly) and chemically stable [9, 10]. It has a wide range of applications, like transparent electrodes, gas sensors, dilute magnetic semiconductors (DMS), window layer for solar cells, active channel layer of transparent thin film transistor (TTFT), photocatalysts, surface acoustic wave devices, microsensors, and photodetectors [11, 12].

Graphene, a flat monolayer of two-dimensional (2D) honeycomb carbon atoms, has a wide range of applications due to its superior structural and electronic properties [13–15]. It can be synthesized by several methods, including micromechanical exfoliation [16], thermal expansion [17], chemical vapor deposition [18], and reduction from GO [19, 20]. Recently, there has been much progress in the self-assembly of nano-colloidal particles for photonics, sensors, supercapacitors, electronics, and other applications. Self-assembly technique provides a facile, rapid, inexpensive, scalable, controllable, and good way to deposit nano- and micrometer-sized particles. Controlling the interactions among particles and particle kinetics is required for device fabrication using the self-assembly method [21–23]. The colloidal composition, concentration, and system setup were considered while performing the experiment (more details about the experiment procedure can be found in the “Experimental” section).

Some studies have primarily focused on ZnO-based photodetectors [24–27]. ZnO-based nanostructured photodetectors exhibited a relatively long response time [28]. However, building an electric field within a heterostructure junction is one of the strategies to separate and transport the photo-carriers [29]. In the open literatures, there are some attempts to introduce GR-ZnO nano-composites, ZnO nanowires, GR arrays/films [30, 31], GR-ZnO nanorods [32, 33], and GR wrapped to hollow ZnO spheres [34]. Moreover, resistance switching of GR to ZnO as a resistive random access memory was reported [35]. However, the reports on the assembly of GO on ZnO films and the application of GO/ZnO heterostructure in UV sensing are still quite rare. Here, a low-cost, facile, and scalable technique was used to cover ZnO films by GO nanosheets. We used ZnO thin films as a template for GO to improve the separation efficiency of photo-generated electron hole pairs upon UV irradiation. This hybrid heterostructure exhibited a repeated fast and uniform response to UV illumination because of the high-transport properties of carbon nanostructures.

## Experimental

**Fig. 1:**
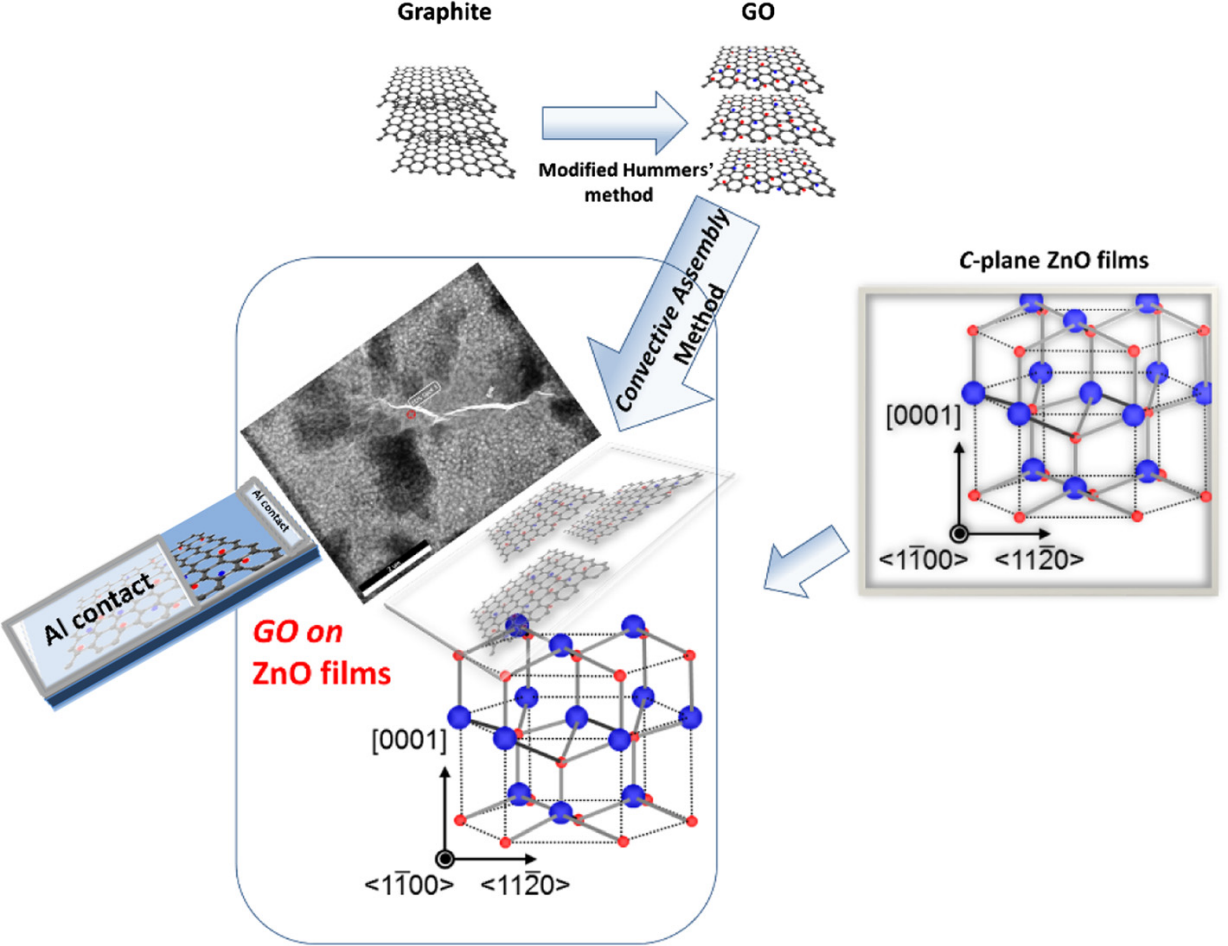
Experimental steps

A schematic diagram for the experimental work is shown in Fig. 1 which can be summarized in three steps. In the first step, 150-nm-thick ZnO thin films were grown on an *a*-plane sapphire substrate using radio-frequency magnetron sputtering technique at deposition temperature of 600 °C, working pressure of 5 × 10^−4^ Torr, background pressure of 2 × 10^−6^ Torr, oxygen fraction of 20 %, and vacuum annealed at 850 °C, as described in references [36, 37]. Smooth *a*-plane sapphire substrates were cleaned using organic solvents, rinsed in DI water, and pre-sputtering of the target was performed to remove contaminations before deposition. In the second step, GO was prepared by chemical oxidation of graphite according to the well-known modified Hummers’ method. A full detailed description of the preparation procedure can be found elsewhere [19, 20]. In the third step, convective assembly technique was used to deposit a continuous thin layer (10 nm) of GO on the prepared ZnO thin films. The convective assembly setup is as follows: a cleaned glass substrate was oriented at 45° with respect to a ZnO film and acts as a knife blade. Eighty microliters of a well-dispersed GO meniscus (1 mg/mL) was injected between the blade and the ZnO film. Then, a slow motion of the blade with a step motor at a speed of 1 cm/h was performed.

Structural characterizations were performed using X-ray diffraction (XRD), field emission scanning electron microscopy (FE-SEM), transmission electron microscopy (TEM), and Raman spectroscopy. Burker-D8 diffractometer with Cuk_α_ radiation was used for XRD measurements. The surface morphology was investigated using FE-SEM (Helios 400). The nano-scale structures were monitored using transmission electron microscopy (model JEM 1230, JEOL, Japan). The characterizations were extended to the lattice vibration modes by micro-Raman spectroscopy measurements at room temperature (model Renishaw System 2000) with Ar^+^ laser at wavelength of 514 nm and power of 3 mW. To evaluate the photoconductivity of the samples, a patterned mask was used to deposit 20-nm-thick Al electrodes (4 mm wide) by thermal evaporation technique as shown in Fig. 1. I-V characteristics were measured at room temperature by a Keithley electrometer (model 6517B). A UV source (254 nm) with power density of 250 mW cm^−2^ was used to irradiate the samples.

## Discussion

**Fig. 2:**
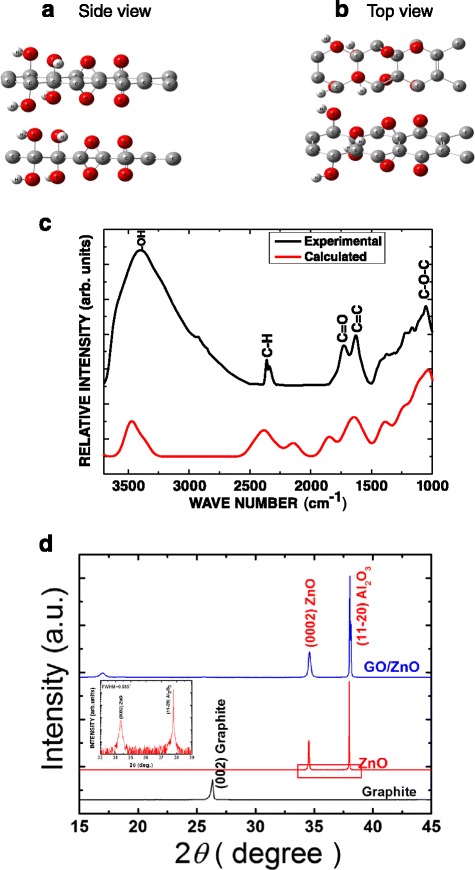
**a**, **b** Top and side view of the proposed GO structure. **c** Comparison of experimental and calculated FTIR spectra. **d** XRD measurements for graphite, ZnO thin films, and assembled GO on ZnO thin films

Direct information about GO structure can be obtained hardly which attracts attention to its structure modeling. Structure is built with Gaussian view program. Structure modeling of GO has been done using Gaussian 03W program. Finally, the output calculations are represented by Chemcraft program as IR spectrum. The following rules were considered while proposing a stable GO structure [38–42]. First, GO contains sp^2^ and sp^3^ orbital hybridization. Second, GO consists of a graphene layer with hydroxyl and carbonyl functional groups. Third, an equal number of the functional groups is built on each side of the graphene sheet. Fourth, each carbon atom cannot be attached with two functional groups. The proposed structure is shown in Fig. 2a (side view) and Fig. 2b (for the top view). Figure 2c shows partial overlap of experimental IR spectrum with the theoretically calculated IR spectrum. The calculations were performed according to the density functional theory (DFT) method using Gaussian 03W program. The peak at 1682.3 cm^−1^ was assigned to the un-oxidized graphitic domains (C = C), and an obvious peak at 3399.0 cm^−1^ was attributed to OH stretching vibrations of the adsorbed water molecules. In addition, the peak at 1725.1 cm^−1^ was assigned to the C = O stretching vibration from the carboxyl and carbonyl groups [19, 39]. Hence, during the oxidation, the original extended conjugated π-orbital of graphite was eliminated and replaced by the oxygen-containing functional groups which settled in the carbon skeleton. This result indicates that the proposed GO structure is close to the synthesized GO structure.

Representative XRD patterns of graphite, ZnO films, and GO on ZnO films are shown in Fig. 2d. For ZnO films on an *a*-plane sapphire substrate, a well-oriented (0002) ZnO peak can be observed beside the reflexes of the substrate. In the inset of Fig. 2d, the symmetric nature, sharpness of the (0002) peak with full width at half maximum (FWHM) of 0.087°, and the absence of reflections from other planes confirm a good *c*-axis orientation perpendicular to the (11–20) plane of the sapphire substrate. The distinct sharp (002) peak of graphite was observed at 2*θ* of 26.55°. After exfoliation, the (002) peak is shifted to a lower angle for GO nanosheets on ZnO films which is related to the increase in the inter-planar spacing beside the reflections of ZnO films and the substrate.

**Fig. 3:**
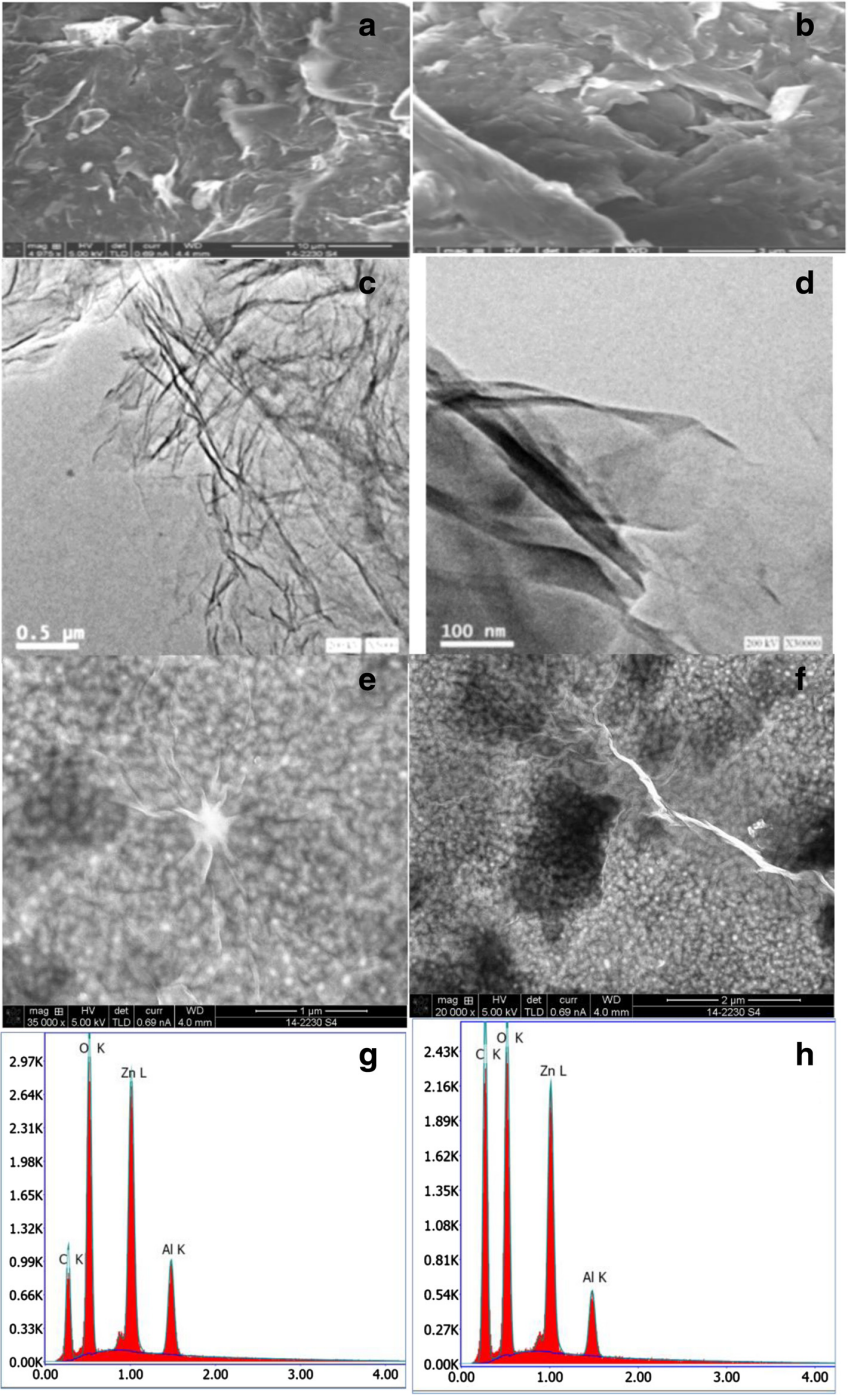
**a**, **b** SEM images of GO nanosheets. **c**, **d** TEM images of GO nanosheets. **e**, **f** FE-SEM images of deposited GO on ZnO films. **g**, **h** EDAX spectra of deposited GO on ZnO films

The surface morphology of GO nanosheets is depicted in Fig. 3a, b. It was found that GO flakes have wrinkles and folds at the edges. Moreover, the shown TEM images in Fig. 3c, d clarify that the synthesized GO nanosheets have few layers with a dimension of few hundred nanometers. The ultra-smooth [36, 37] and well-oriented ZnO thin films with root mean square roughness of 0.3 nm act as a template for the assembled GO nanosheets. On the other hand, after the deposition of GO layer on the template, a detectable continuous, wide area and slightly stacked GO nanosheets were observed (see Fig. 3e, f). The chemical composition of the assembled GO/ZnO/Al_2_O_3_ has been elucidated using EDAX. Only elements of C, Zn, O, and Al can be observed in Fig. 3g, h, which confirm the purity and quality of the prepared samples.

**Fig. 4:**
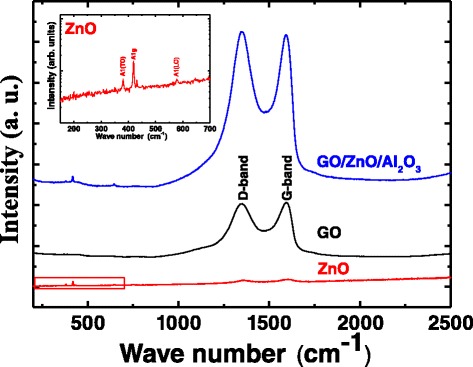
Raman spectrum of ZnO thin films, GO nanosheets, and GO on ZnO thin films. *Inset* magnifies the shown range for ZnO films

Micro-Raman measurements have been carried out to investigate the quality of the prepared samples. Figure 4 represents the room temperature Raman spectrum of ZnO films, GO nanosheets, and GO on ZnO films. It is very clear from the figure that the Raman spectrum of GO exhibited two peaks at 1595 cm^−1^ and 1352 cm^−1^, respectively. Conventionally, the peak at 1352 cm^−1^ is attributed to the defects, and disorders arise in the sp^2^ carbon rings (D-band). The other peak around 1595 cm^−1^ is due to the scattering of the first-order phonons (*E*_2g_) which are usually called G-bands [43, 44]. The inset of Fig. 4 magnifies the shown range of Raman spectra for ZnO films. One can see that ZnO transverse optical mode A1(TO) and longitudinal optical mode A1(LO) appeared at 380 and 576 cm^−1^, respectively [45, 46]. The other peak is at 417, and 650 is attributed to the Al_2_O_3_ substrate [47, 48]. After the assembly of GO on ZnO, in addition to the G- and D-bands of GO, the characteristic ZnO and sapphire modes can be observed. However, the intensity ratio between the G-band and the D-band (*I*_G_/*I*_D_) has been changed from 1.01 for GO alone to 0.99 after the assembly of GO on ZnO films. The small change in the ratio implies that there is no additional defect introduced in the case of GO/ZnO [49].

**Fig. 5:**
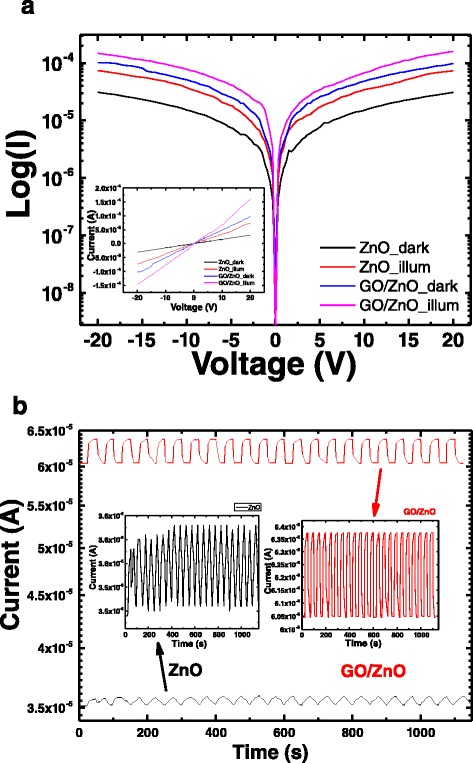
**a** Log plot of I-V characteristics for ZnO films and GO/ZnO in dark and in the presence of UV illumination. *Inset* represents the linear ohmic behavior. **b** UV on-off test for ZnO (*black line*) and GO/ZnO (*red line*)

We exposed ZnO and GO/ZnO heterostructures to UV illumination, and the output currents were recorded using the Keithley electrometer as a function of applied voltage. All the samples follow ohmic behavior and the current increase with increasing the applied bias as shown in a linear plot (inset of Fig. 5a) and an algorithmic plot (Fig. 5a). Under UV illumination, a dramatic increase of current was recorded. The generated photocurrent of GO/ZnO was compared with that of ZnO films. Upon irradiation of the heterostructure, the increase in current can be explained in terms of photo-generated electrons which were collected and transported through the paths provided by GO nanosheets since carbon nanostructures have high electron acceptor ability and improve the electron transport properties [50]. Therefore, electrons move from the valance band to the conduction band of ZnO, finding a transport path through GO nanosheets [51]. In order to confirm the feasibility of the prepared heterostructure for UV sensing applications, an on-off test was measured. The applied bias during on/off was 10 V. With alternating switch on and switch off events, a fast and stable photocurrent was detected in the case of the heterostructure (Fig. 5b). In comparison, there was no similar response of ZnO films. Electron hole pairs are generated when the UV illumination is on, and the generated carriers are easily transported through the heterostructure. On the other hand, electron hole pairs recombine quickly when UV illumination is turned off. The recovery speed of the heterostructure is much faster than ZnO films, and the generated photocurrent by GO/ZnO heterostructure was about two times as high as that of ZnO films. Moreover, response current (*I*_ph_) was used to calculate the on-off ratio of photocurrent where *I*_ph_ = *I*_UV_−*I*_d_, where *I*_d_ is the current in dark and *I*_UV_ is the current under UV illumination condition [33]. The on-off ratio of photocurrent is defined as *I*_ph_/*I*_d_. The photocurrent on-off ratio of heterostructure and ZnO films were 0.055 and 0.016, respectively. It is well known that UV sensing of ZnO is related to the absorption and desorption of oxygen molecules on ZnO surface and GO nanosheets enhance the carrier transport [30]. Additionally, the device responsivity *R*_s_ can be calculated from the following equation: *R*_s_ = (*I*_ph_/*P*_o_*A*), where *P*_o_ is the UV power density, A the active area, and *I*_ph_ the response current. The calculations were extended to the external quantum efficiency of the photodetector which can be calculated by $\mathrm{E}\mathrm{Q}\mathrm{E} = \frac{R_{\mathrm{s}}\times h\vartheta \kern0.5em }{e} \times 100$, where *hυ* is the energy of the incident photon [52]. *R*_s_ and EQE for ZnO heterostructure were 37 × 10^−4^ A/W and 1.8 %, respectively, while their values were only 6.2 × 10^−4^ A/W and 0.3 % for ZnO films. Therefore, the UV photo-response performance of GO/ZnO heterostructure is much higher than ZnO films. The obtained *R*_s_ value is consistent with the data reported for graphene sheet-based photodetectors (0.1 ~ 0.5 mA/W) [53].

## Conclusion

Surface functionalization of ZnO films by the GO layer was conducted by self-assembly technique. ZnO films act as a good template for the deposited GO layer because of its smoothness. It is worth noting that we emphasized the enhancement in the UV photo-response performance for GO/ZnO heterostructure with respect to ZnO films. Since GO creates two-dimensional electronic-conducting channels for the photo-generated carriers, separation and transport of photo-generated electron hole pairs and reducing the recombination improve the on-off ratio.
